# Differently expressed miRNA in plasma samples of immune thrombocytopenic purpura patients and its clinical significance

**DOI:** 10.5937/jomb0-52498

**Published:** 2025-08-21

**Authors:** Yuqian Yao, Hemeng Zhao, Xiaoyu Zhu, Yafei Fang, Yue Feng

**Affiliations:** 1 The First People's Hospital of Lianyungang & Xuzhou Medical University Affiliated Hospital of Lianyungang & The First Affiliated Hospital of Kangda College of Nanjing Medical University, Department of Pediatric Internal Medicine, Lianyungangk, People's Republic of China

**Keywords:** miRNAs, immune thrombocytopenic purpura, receiver operator characteristic, clinical value, expression, miRNA, imunska trombocitopenijska purpura, ROC kriva, klinička vrednost, ekspresija

## Abstract

**Background:**

To guide clinical doctors, current work has been designed to explore the abnormal expressions of miRNAs in plasma samples of patients with immune thrombocytopenic purpura (ITP).

**Methods:**

Bioinformatic analysis was performed using the GSE80401 chip. The study subjects were recruited from the First People's Hospital of Lianyungang between May 2021 and December 2023. 48 ITP patients admitted to the intensive care unit were enrolled. miRNA levels were examined using real-time polymerase chain reaction. All data were analysed using SPSS 22.0 software. The potential diagnosis value of the significantly up-regulated and down-regulated miRNAs was evaluated using a receiver operator characteristic (ROC) curve.

**Results:**

We performed bioinformatical analysis on the GSE80401 chip and identified the differently expressed miRNAs in ITP patients compared to the controls, and the results of the heat map showed the results. GO and pathway analysis revealed the process that involved the differently expressed miRNAs. Next, the results of RT-qPCR analysis showed the levels of miR-877-3p, miR-425-3p, miR-122-5p, miR-1281, and miR-1825 were significantly increased. In contrast, the miR-3945, miR-4430, miR-3158-5p, miR-3131, and miR-4655-3p levels were markedly decreased in plasma samples of ITP patients compared with the controls. Finally, results showed that the area under the ROC curve of miRNAs was as follows: miR-877-3p, 0.9349, miR-425-3p, 0.8607, miR-1281, 0.7131, miR-1825, 0.8928, miR-3945, 0.8459, miR-4430, 0.8112, miR-3158-5p, 0.6059, miR-3131, 0.8989, suggesting that the above miRNAs may serve as biomarkers for distinguishing the ITP patients from healthy controls.

**Conclusions:**

miRNAs may have predictive value for the diagnosis of ITP. The results of current work may provide new clues for the pathogenesis of ITP, which in turn offers a new theoretical basis and therapeutic tools for the clinical diagnosis and treatment of ITP.

## Abstract (sr)

**Uvod:**

Cilj ovog rada bio je da istraži abnormalnu ekspresiju miRNA u uzorcima plazme pacijenata sa imunskom trombocitopenijskom purpurom (ITP) kako bi se pružile smernice kliničarima.

**Metode:**

Bioinformatička analiza je sprovedena korišćenjem čipa GSE80401. Ispitanici su regrutovani iz Prve narodne bolnice u Lianyungangu između maja 2021. i decembra 2023. godine. U studiju je uključeno 48 pacijenata sa ITP primljenih na jedinicu intenzivne nege. Nivoi miRNA su ispitivani metodom lančane reakcije polimeraze u realnom vremenu (RT-qPCR). Svi podaci analizirani su pomoću softvera SPSS 22.0. Potencijalna dijagnostička vrednost značajno pojačano i smanjeno eksprimiranih miRNA je procenjena korišćenjem ROC (receiver operator characteristic) krive.

**Rezultati:**

Sproveli smo bioinformatičku analizu na čipu GSE80401 i identifikovali različito eksprimirane miRNA kod pacijenata sa ITP u poređenju sa kontrolnom grupom, pri čemu su rezultati prikazani pomoću toplotne mape. GO analiza i analiza puteva pokazale su procese u koje su uključene različito eksprimirane miRNA. Dalje, rezultati RT-qPCR analize su pokazali da su nivoi miR-877-3p, miR-425-3p, miR-122-5p, miR-1281 i miR-1825 značajno povećani, dok su nivoi miR-3945, miR-4430, miR-3158-5p, miR-3131 i miR-4655-3p bili značajno smanjeni u uzorcima plazme pacijenata sa ITP u poređenju sa kontrolnim učesnicima. Na kraju, rezultati su pokazali da je površina ispod ROC krive za miRNA iznosila: miR-877-3p, 0.9349, miR-425-3p, 0.8607, miR-1281, 0.7131, miR-1825, 0.8928, miR-3945, 0.8459, miR-4430, 0.8112, miR-3158-5p, 0.6059, miR-3131, 0.8989, što sugeriše da navedene miRNA mogu da posluže kao biomarkeri za razlikovanje pacijenata sa ITP od zdravih kontrola.

**Zaključak:**

miRNA mogu imati prediktivnu vrednost za dijagnozu ITP. Rezultati ovog rada mogu pružiti nove smernice za razumevanje patogeneze ITP, što dalje pruža teorijsku osnovu i terapijske alate za kliničku dijagnozu i lečenje ITP.

## Introduction

Immune thrombocytopenic purpura (ITP) is one of the most diagnosed diseases in childhood caused by abnormal T cell reactions, accounting for approximately 30% of all hemorrhagic diseases [1] [2]. It is characterised by a decreased count of peripheral blood platelets and a normal or increased count of bone marrow megakaryocytes accompanied by maturation disorder [3]. Statistics show that the annual incidence of ITP in children is 4.8 per 100,000 [4], and ITP accounts for 25.1% of all bleeding disorders in children [5]. Younger adult patients are predominantly females, but the prevalence of ITP in older patients is essentially the same in men and women [6] [7]. The primary therapies include glucocorticoids, immunosuppressants, splenectomy, and other nonspecific methods, but the side effects are apparent and seriously affect the patient's quality of life [3] [8].

Recent research has shown that abnormal cellular immune function may participate in ITP pathogenesis [9]. MicroRNAs (miRNAs) are endogenous small RNA molecules containing about 20-22 nucleotides [10], and they pair with specific messenger RNAs (mRNA), causing mRNA degradation that regulates the translation of proteins after transcription [11]. miRNAs have evolved to be highly conserved in phylogenetically close species and have become potent regulators of cell growth, cell differentiation and cancerous [12]. Moreover, miRNAs were abnormally expressed in various cancers [13] [14] [15]. Prior research suggests that mi RNA disorders are associated with autoimmune diseases [16].

Microarray testing of plasma samples from patients with ITP and healthy controls has found increased miRNA levels in patients with ITP compared with healthy individuals [17]. The roles of miRNAs in ITP have been discussed. For example, Garabet et al. suggested that miR-199a-5p could discriminate patients with ITP from healthy controls and may serve as a diagnostic marker for ITP [18].; moreover, it has been reported that miR-641 may play important roles in ITP through regulating Th17/Treg balance [19]. Nevertheless, studies on the mechanism of miRNAs in ITP remain in the initial stage. Accordingly, we conducted a study investigating miRNA levels in ITP to guide clinical doctors.

## Materials and methods

### Bioinformatics analysis

GSE80401 was analysed using Geoexplorer (https://geoexplorer.rosalind.kcl.ac.uk/) and GE02R (https://www.ncbi.nlm.nih.gov/geo/geo2r/) as previously described. Gene ontology (http://www.geneongoloty.org/) and DAVID 6.8 (https://david.ncifcrf.gov/) have been applied for GO and KEGG analysis. A P-value less than 0.05 has been considered as significant.

### Patients

The study subjects were recruited from the First People's Hospital of Lianyungang between May 2021 and March 2023. Forty-eight ITP patients admitted to the intensive care unit were finally enrolled. Forty-eight healthy subjects were also enrolled as the healthy control group. Current work has been approved by The First People's Hospital of Lianyungang (No. 202104314) ethical committee, and an informed consent form has been acquired from all patients.

### Plasma RNA isolation

Blood samples from the ITP patients and healthy controls have been isolated and maintained using EDTA pre-treated tubes. The samples were then centrifuged (200 g, 10 mins), the supernatants were transferred, and then centrifuged (16,000 g, 10 mins). Next, RNAs were isolated using low platelet plasma (300 μL) by the kit provided by Beyotime (Shanghai, China). Briefly, lysis buffer was added to the blood samples, and then an equal volume of binding buffer was added and mixed. Then, the mixture was transferred to the columns and centrifuged at 12,000xg for 30 seconds. The liquid was then discarded, 600 μL washing buffer I was added, and the tube was centrifuged for 30 seconds (12,000 xg); then 600 μL of washing buffer II was added, and the tune was centrifuged for 30 seconds (12,000xg). This step was repeated, and then the samples were centrifuged at the highest speed for 2 minutes; then, the samples were placed in an RNA elution tube, and 30-50 μL elution buffer was added. The samples were then kept at room temperature for 2 minutes and centrifuged at the highest speed for 30 seconds. The total RNAs were obtained, and the samples were stored at -80°C until needed.

### RT-qPCR

The treated total peripheral blood RNA was extracted by TRIzol reagent (Invitrogen), and cDNAs were synthesised by BeyoRT™ II cDNA Kit (Beyotime). PCR has been performed using Easy-Load™ PCR Master Mix (Beyotime) to detect miRNA expressions. The PCR condition was 95 for 30 sec, then 40 cycles of 94°C for 15 sec and 60°C for 30 sec. The 2—AACt method has been applied for quantification, and U6 was used for internal references. The sequences of the primers were as follow: miR-877-3p F, 5'-CGTGTGTCCTCTTCTCCCTCC-3', R, 5'-AGTGCAGGGTCCGAGGTATT-3'; miR-425-3p F, 5'-CGTGTCCGCCCAGTGC-3', R, 5'-AGTGCAGGGTCCGAGGTATT-3'; miR-122-5p F, 5'-GTATGATGGAGTGTGACAA-3', R 5'-TGGTGTCGTGGAGTCGT-3'; miR-1281 F, 5'-GCGCGTCGCCTCCTCC-3', R, 5'-AGTGCAGGGTCCGAGGTATT-3'; miR-1825 F, 5'-CGCGTCCAGTGCCCTC-3', R, 5'-AGTGCAGGGTCCGAGGTATT-3'; miR-3945 F, 5'-CGAGGGCATAGGAGAGGGT-3', R, 5'-AGTGCAGGGTCCGAGGTATT-3'; miR-4430 F, 5'-GCGCGAGGCTGGAGTGA-3', R, 5'-AGTGCAGGGTCCGAGGTATT-5'; miR-3158-5p F, 5'-GCGCCTGCAGAGAGGAAG -3', R, 5'-AGTGCAGGGTCCGAGGTATT-3'; miR-3131 F, 5'-CGTCGAGGACTGGTGGAAG-3', R, 5'-AGTGCAGGGTCCGAGGTATT-3'; miR-4655-3p F, 5'-GCGACCCTCGTCAGGTCC-3', R, 5'-AGTGCAGGGTCCGAGGTATT-3'; U6, F, 5'-AAAGCAAATCATCGGACGACC-3', R, 5'-GTACAACACATTGTTTCCTCGGA-3'.

### Statistics

Data has been evaluated by SPSS22.0 software, and data is expressed as mean ± SD. Students' t-test tested differences between two groups, while differences among >2 groups were tested by one-way analysis of variance, as with Turkey's post-hoc test. The P-value less than 0.05 has been considered a significant difference.

## Results

### Differentially expressed miRNAs in ITP

**Figure 1:**
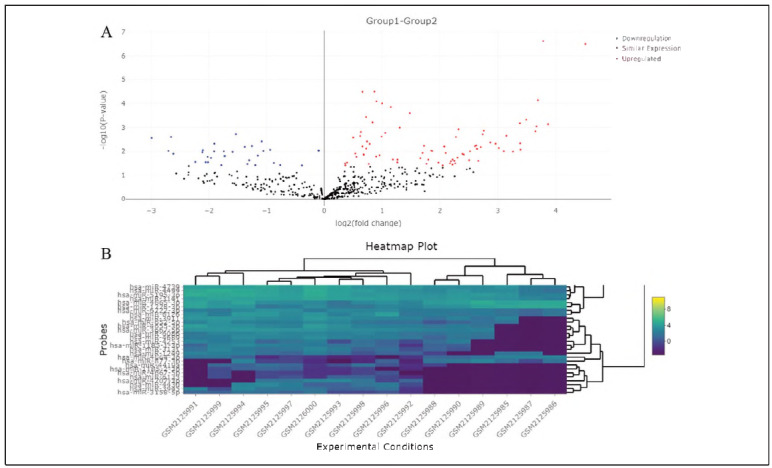
Differentially expressed miRNAs in ITP. A. results of volcano plots. B. results of heat map.

First, bioinformatic analysis based on GSE80401 has been conducted to compare miRNA expressions in ITP (n = 10) and the control group (n = 6). The results of volcano plots showed the significantly up-regulated (red dots) or down-regulated (blue dots) miRNAs between ITP and control groups (Figure 1A). Additionally, results of the heat map showed the most significantly up-regulated and down-regulated mlRNAs between ITP (n = 10) and control group (Figure 1B).

### GO and pathway analysis

**Figure 2:**
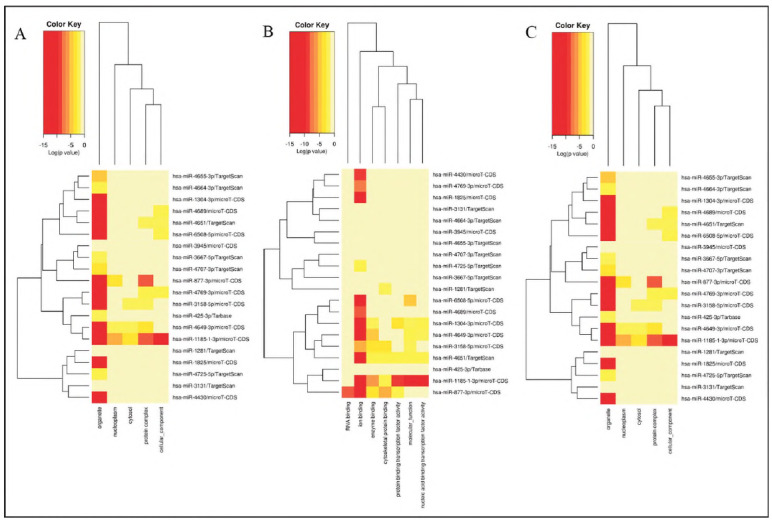
Results of GO analysis. A. Biological process. B. Cellular component. C. Molecular function

Next, the function of the differently expressed mlRNAs in ITP was evaluated using GO analysis and KEGG pathway analysis. For the biological process GO terms, including PC-gamma receptor signaling pathway involved in phagocytosis, cellular protein modification process, cellular nitrogen compound metabolic process, biosynthetic process, gene expression, catabolic process, small molecule metabolic process, neurotrophln TRK receptor signaling pathway, Fc-epsilon receptor signaling pathway, cellular protein metabolic process, post-translational protein modification, blood coagulation, cellular component assembly, macromolecular complex assembly, phosphatidylinositol-mediated signaling, epidermal growth factor receptor signaling pathway, synaptic transmission, fibroblast growth factor receptor signaling pathway, mitotic cell cycle, DNA metabolic process, cell death, response to stress, transcription, DNA-templated, biological process (Figure 2A); for the cellular component GO terms, including Organelle, nucleoplasm, cytosol, protein complex, cellular component (Figure 2B); for the molecular function GO terms, including RNA binding, Ion binding, enzyme binding, cytoskeletal protein binding, protein binding transcription factor activity, molecular function, nucleic acid binding transcription factor activity (Figure 2C).

**Figure 3:**
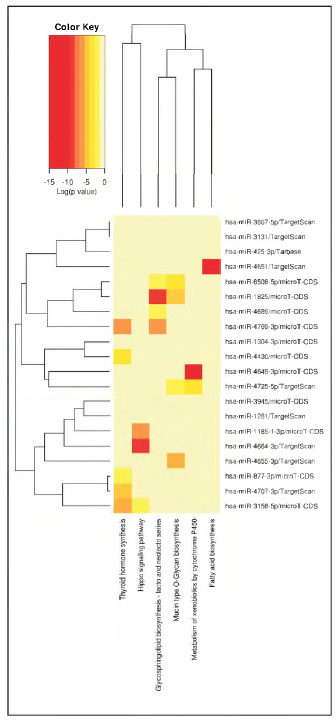
Results of KEGG pathway analysis.

Moreover, results of KEGG pathway analysis suggested the differentially expressed mRNAs were enriched in several pathway (Figure 3), including thyroid hormone synthesis, Hippo signaling pathway, glycosphlngolipid biosynthesis-lacto and neolacto series, mucin type O-Glycan biosynthesis, metabolism of xenobiotics by cytochrome P450, fatty acid biosynthesis.

### Comparison of the differently expressed mlRNAs between ITP patients as well as healthy controls

**Figure 4:**
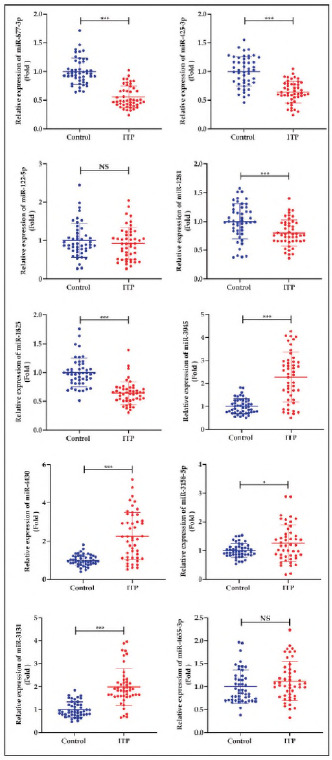
Comparison of the up-regulated and down-regulated miRNAs between ITP patiens and healthy controls.<br>*p<0.05, **p<0.01, ***p<0.001

Furthermore, we collected the plasma samples of patients with ITP as well as the controls, expressions of the top 5 over-expressed as well as decreased mlRNAs have been compared. As shown In Figure 4, expressions of mlR-877-3p, mlR-425-3p, mlR-122-5p, mlR-1281 as well as mlR-1825 were significantly increased, while the levels of miR-3945, miR-4430, miR-3158-5p, miR-3131 as well as miR-4655-3p were markedly decreased in plasma samples of the ITP patients in comparison with the healthy controls (p<0.05). Meanwhile, levels of miR-122-5p as well as miR-4655-3p did not markedly changed (p>0.05).

### Potential diagnostic value of the up-regulated as well as down-regulated miRNAs

**Figure 5:**
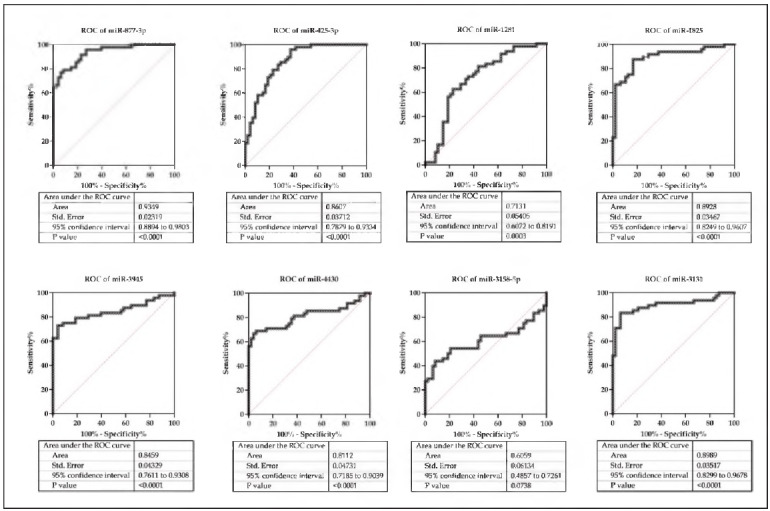
ROC curve for the up-regulated and down-regulated miRNAs.

The potential diagnosis value of the significantly changed miRNAs has been evaluated by drawing the ROC curve. We found the AUC of miRNAs were as follow: miR-877-3p, AUC 0.9349, 95% confidence Interval (Cl) 0.8894 to 0.9805; mlR-425-5p, AUC 0.8607, 95% Cl 0.7879 to 0.9554; mlR-1281, AUC 0.7151, 95% Cl 0.6072 to 0.8191; mlR-1825, AUC 0.8928, 95% Cl 0.8249 to 0.9607; mlR-5945, AUC 0.8459, 95% Cl 0.7611 to 0.9508; mlR-4450, AUC 0.8112, mlR-5158-5p, AUC 0.6059, 95% Cl 0.4857 to 0.7261; mlR-5151, AUC 0.8989, 95% Cl 0.8299 to 0.9678 (Figure 5).

## Discussion

ITP Is an autoimmune disease of the children, which Is prone to develop Into refractory ITP [1]. ITF) especially refractory ITF) brings a heavy mental and economic burden to patients and their families. Further exploration of Its pathogenesis Is of great significance to Improve the long-term life quality of ITP patients and their families [20]. The pathogenesis of ITP remains unclear, but It Is certain that Immune factors play a dominant role [2]. miRNAs, I.e. mlR-146a as well as mlR-155, play key roles during the process of the occurrence as well as development of autoimmune diseases [21] [22]. In current work, through blolnformatlc analysis of the GSE80401 chip, we successfully Identified the differently expressed miRNAs between patients with ITP as well as the controls. We have Identified the top five Increased miRNAs (mlR-877-5p, mlR-425-5p, mlR-122-5p, mlR-1281 and mlR-1825) and the top five decreased miRNAs (mlR-5945, mlR-4450, mlR-5158-5p, mlR-5151 and mlR-4655-5p). In the ROC analysis, the differently expressed miRNAs have shown clinical predictive value for ITP. GO and pathway analysis showed the process that the diffusely expressed miRNAs Involved.

miRNAs may severe as Important regulators In the development of Immune cells, regulation of Immune responses and many autoimmune diseases through the regulation of their target genes [23]. The expression profile of T-cell miRNAs In ITP patients were different In comparison with the normal controls [16]. In current study, we have successfully the differently expressed miRNAs In ITP by blolnformatlc analysis of the GSE80401 chip. Besides, results of GO analysis as well as KEGG pathway analysis Indicated the biological process, cellular component, molecular function and signaling pathways these miRNAs may be Involved. Interestingly, most of the GO terms were strongly associated with the process of ITF) and for the KEGG pathway analysis, the relationship between thyroid hormone synthesis [24], mucin type O-Glycan biosynthesis [25], metabolism of xenoblotlcs by cytochrome P450 [26], fatty acid biosynthesis [27] [28] and ITP have also been discussed. Therefore, based on the results of bioinformatic analysis, we can conclude that miRNAs were strongly associated with the development of ITP. However, the underlying mechanism still requires further investigation.

Reduced peripheral blood platelets, increased or normal numbers of bone marrow megakaryocytes with impaired maturation and the detection of anti-platelet autoantibodies in the body are the main features of ITP [29]. The body produces auto-platelet antibodies against its own platelet membrane surface glycoproteins. The sensitised platelets are engulfed by the reticuloendothelial system, ultimately leading to thrombocytopenia [30]. As precursor platelet-producing cells, megakaryocytes also express platelet membrane glycoproteins on their surface, and platelet autoantibodies act on megakaryocytes to increase their destruction or impair their development [31]. Megakaryocytes is mediated by multiple factors, while the abnormalities in any one of them may lead to impaired megakaryocytogenesis. Transcription factors play a key role in megakaryocyte development [32] [33]. miRNAs can regulate megakaryocytogenesis by regulating the expression of key transcription factors involved in megakaryocyte development. miRNAs molecules regulate megakaryocyte development by individually or in combination with the expression of their target genes. In turn, impaired megakaryocyte development is an important pathogenetic mechanism of ITP [34] [35]. Therefore, we speculate that miRNAs could regulate the procession of ITP through the regulation of megakaryocyte development.

ROC curves have been applied for assessing the potential diagnostic value of different diseases [36]. In current study, results of ROC analysis declared the potential diagnostic value of the up-regulated miRNAs and miR-577-5p has the highest AUC (0.9549) for the top 5 up-regulated miRNAs tested alone. Among the top5 down-regulated miRNAs, miR-5151 had an AUC of 0.8989. These results suggest that miRNAs testing may have predictive value in the diagnosis of ITP.

This study has certain limitations. Due to the limited number of ITP patients admitted to our hospital each year, we were able to collect only 48 samples. Future studies should include a larger sample size to strengthen the conclusions drawn from the current research.

## Conclusion

This study explores miRNAs in ITP, providing new clues for the pathogenesis of ITP, which in turn offers new theoretical basis and therapeutic tools for the clinical diagnosis and treatment of ITP.

## Dodatak

### Acknowledgements

Not applicable.

### Funding

Haosen Foundation for Young Talents of the First People's Hospital of Lianyungang City(QN1705).

### Availability of data and materials

Not applicable.

### Authors' contributions

Yue Feng contributed to the conception of the study and performed the experiment.

Yafei Fang contributed significantly to analysis and manuscript preparation.

Xiaoyu Zhu performed some experiments and revised the manuscript.

Yuqian Yao performed the data analyses and wrote the manuscript.

Hemeng Zhao helped perform the analysis with constructive discussions.

### Ethics approval and consent to participate

This study protocol was approved by the Ethics Committee of The First People's Hospital of Lianyungang. Written informed consent was provided prior to the study.

### Patient consent for publication

Not applicable.

### Conflict of interest statement

All the authors declare that they have no conflict of interest in this work.
